# Arrhythmogenic right ventricular cardiomyopathy with sustained ventricular tachycardia: a case report

**DOI:** 10.1186/s12872-024-03959-z

**Published:** 2024-05-30

**Authors:** Ying Ban, Feng-juan Yao, Wei Li

**Affiliations:** Department of Medical Ultrasonics, The First Affiliated Hospital of Sun Yat-Sen University, 58 Zhongshan Road 2, Guangzhou, 510080 People’s Republic of China

**Keywords:** Arrhythmogenic right ventricular cardiomyopathy, Myocardial contrast echocardiography, Myocardial fibrosis

## Abstract

**Introduction:**

Arrhythmogenic right ventricular cardiomyopathy (ARVC) is an infrequent hereditary disorder distinguished by fibrofatty replacement of the myocardium in the right ventricular, which predisposes individuals to life-threatening arrhythmias. This case delineates an ARVC patient who suffered recurrent bouts of sustained ventricular tachycardia (VT). In this case, we mainly discuss the application of myocardial contrast echocardiography (MCE) in displaying myocardial fibrosis in patients with ARVC.

**Case presentation:**

A 43-year-old male experienced three episodes of unexplained VT over an eight-year period, accompanied by symptoms of chest discomfort, palpitations and dizziness. Coronary angiography revealed no significant coronary stenosis. The electrocardiogram (ECG) results indicated characteristic epsilon waves in right precordial leads, and subsequent echocardiography identified right ventricular enlargement and right ventricular systolic dysfunction. MCE further disclosed regional myocardial ischemia at the epicardium of the left ventricular apex. Ultimately, cardiovascular magnetic resonance imaging (CMR) corroborated the ARVC diagnosis, highlighting linear intensification in the right ventricle during the delayed enhancement.

**Conclusion:**

Prompt identification of ARVC is crucial for timely intervention and management. MCE may offer an effective and valuable technique for the detection of myocardial involvement in ARVC patient.

## Introduction

Arrhythmogenic right ventricular cardiomyopathy (ARVC) is a hereditary condition characterized by the progressive replacement of myocardial tissue with fibrofatty deposits, predominantly affecting the right ventricle (RV), predisposing patients to fatal arrhythmias and resulting in a slow progression of ventricular dysfunction [1]. The disorder exhibits a gender disparity, favoring male patients with an approximate ratio of 3:1 [2]. Clinical presentation of ARVC is diverse, prominently marked by episodes of arrhythmias, syncope and even sudden cardiac death (SCD). Notably, the primary clinical feature often consists of recurrent and sustained ventricular tachycardia (VT) [3], serving frequently as the initial manifestation of this cardiomyopathy.

## Case report

A previously healthy 43-year-old male patient presented with acute chest discomfort and palpitations subsequent to alcohol ingestion, with these symptoms escalating to syncope. The electrocardiogram (ECG) delineated VT with a heart rate (HR) of 230 beats per minute (bpm). The patient’s consciousness recovered after receiving medication treatment and subsequently radiofrequency ablation (RFA) was performed. However, identical symptomatology reemerged after further alcohol consumption a month later, while the ECG did not suggested VT and the second RFA endeavor was not successfully induced VT. Since the patient received care at other medical institutions for his initial two episodes of arrhythmia, the prescribed medication regimen post-discharge was not accessible. No further incidents were reported in the next eight years until the patient again experienced chest constriction and palpitations post-alcohol consumption one week ago, with an ECG verifying VT at a HR of 193 bpm. Ongoing hemodynamic instability necessitated cardioversion to restore sinus rhythm. Subsequent coronary angiography indicated the presence of a coronary myocardial bridge and slow blood flow in the right coronary artery, without identifying significant plaques or stenoses in the coronary artery main branches.

**Fig. 1:**
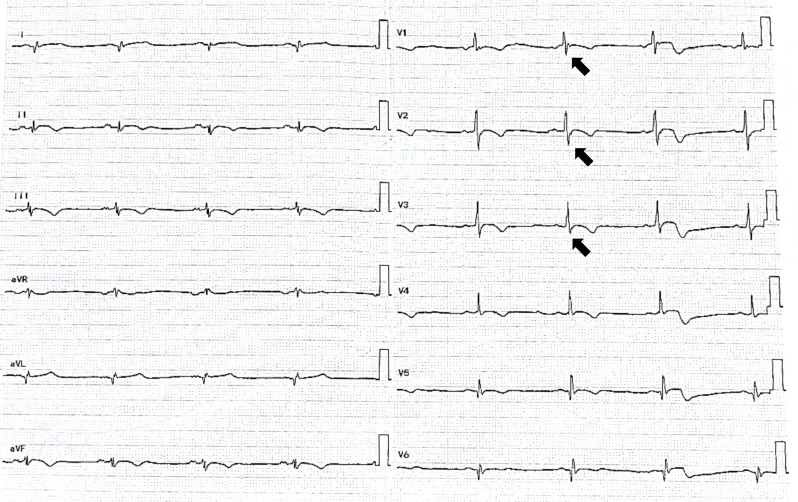
The ECG demonstrated epsilon waves in V1-V3 leads

**Fig. 2:**
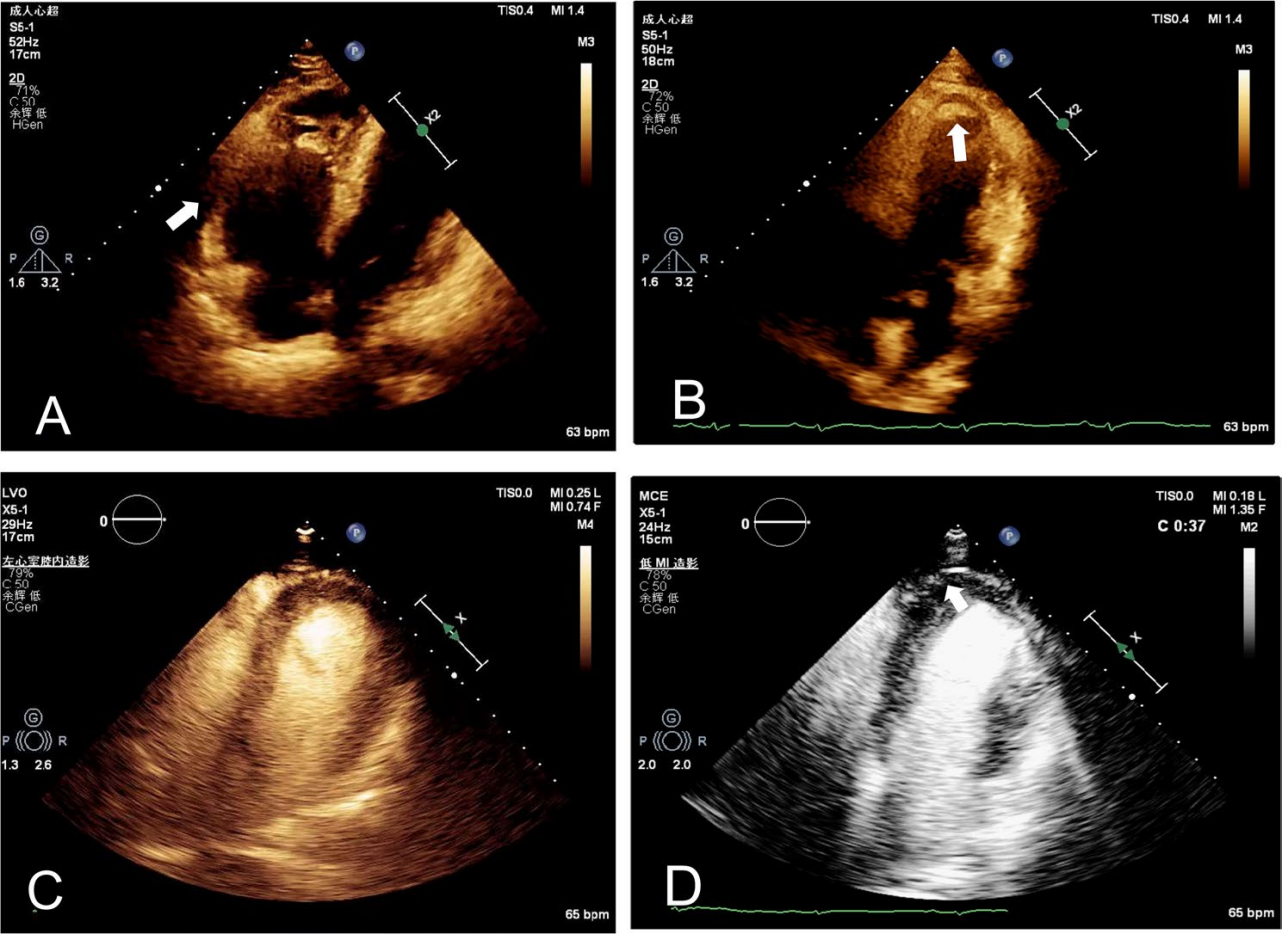
**A**, Echocardiography showed right ventricular enlargement with thinning of the ventricular walls and reduced motion of the right ventricular wall; **B**, Echocardiography showed a flake isoechoic lesion in the apex of the LV; **C**, LVO reveals no apparent filling defect at the apex and reduced motion in the anterior, lateral, inferior segments of the left ventricular apex; **D**, MCE patten shows markedly lower perfusion levels of the subepicardial myocardium at the left ventricular apex compared to the endocardial enhancement at the same level

**Fig. 3:**
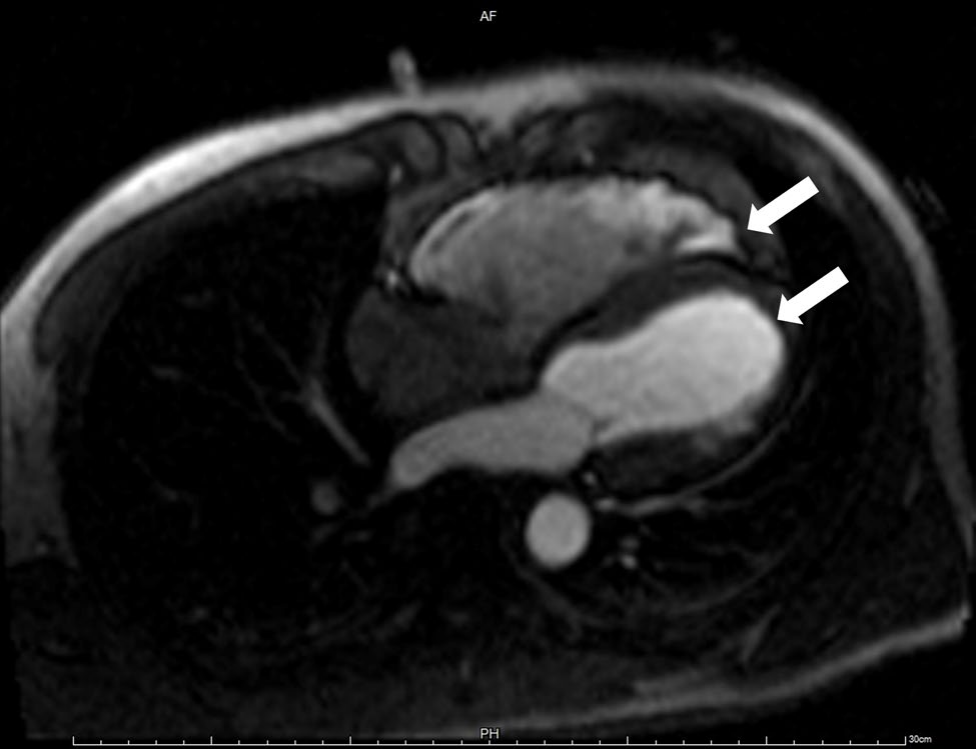
Delayed enhancement in CMR demonstrates subendocardial diffuse patchy linear enhanced lesions in the left ventricular apex-middle free wall

The patient was referred to our hospital to investigate the cause of the recurrent episodes of VT. Physical examination revealed no abnormalities and laboratory tests were within normal range, barring mild elevations in of high-sensitive troponin T (TnT) and procalcitonin (PCT). Post-admission ECG unveiled sinus bradycardia, with biphasic P wave modifications in lead II suggestive of left atrial enlargement, T-wave inversions in multiple leads, and the presence of pathological Q waves in leads I and AVL. Intriguingly, epsilon waves were detected in leads V1-V3 (Fig. 1). Two-dimensional echocardiography showed considerable RV dilation with ventricular wall thinning and diminished motion (Fig. 2A). Additionally, a flake isoechoic lesion was observed in the left ventricle (LV) apex and the movement of LV apex accordingly decreased as well (Fig. 2B). Contrast echocardiography was performed to ascertain the presence of a thrombus in the LV but no filling defect was observed on left ventricular opacification (LVO) (Fig. 2C). However, myocardial contrast echocardiography (MCE) revealed a notable reduction in apical epicardial perfusion (Fig. 2D). Cardiovascular magnetic resonance imaging (CMR) elucidated the linear intensification on the delayed enhanced scan of the RV and the subendocardial diffuse stripy late gadolinium enhancement (LGE) in the left ventricular apex-middle free wall (Fig. 3).

Ultimately, the diagnosis of ARVC was confirmed, characterized by diffuse transmural fibrosis in the right ventricular free wall and subepicardial myocardial fibrosis in the left ventricular apex free wall. Due to the patient’s decision against the implantation of an intracardiac defibrillator (ICD), he underwent a RFA again and VT was successfully induced during the operation, which originated from the base of RV and the free wall of the tricuspid annulus. Following treatment, the patient was discharged with prescribed verapamil with plans for subsequent follow-up.

## Discussion

Identifying ARVC presents considerable challenges, stemming from its insidious onset and nonspecific clinical manifestations. Moreover, a definitive diagnosis of ARVC is rarely achieved through any single standard diagnostic method; instead, it relies on a qualitative scoring system that incorporates various aspects. The 1994 International Task Force (ITF) initially included major and minor criteria based on a comprehensive combination of defects in RV morphology and function, depolarization/repolarization ECG abnormalities, tissue pathology, typical arrhythmias, family history, and genetic testing results. The identification of myocardial fibrofatty replacement through endomyocardial biopsy (EMB) was considered pivotal according to the 1994 ITF criteria [4]. The ITF criteria revisions in 2010 introduced quantitative measures for diagnosing structural and functional RV abnormalities [5]. Furthermore, in 2009, Asimaki et al. proposed a new diagnostic approach, suggesting that histologic evidence of fibrofatty replacement of the ventricular myocardium with a subepicardial mid-mural or transmural involvement, in the absence of obstructive atherosclerotic plaques within the corresponding coronary artery, as a diagnostic criterion for ARVC [6].

Myocardial fibrosis, the primary pathological feature of ARVC, manifests microscopically as abnormal proliferation of fibroblasts, excessive collagen deposition, and irregular distribution [7]. This fibrotic progression stealthily advances, laying the groundwork for a spectrum of cardiovascular pathologies. In terms of electrophysiology, the presence of non-conductive fatty tissue around myocardial fibers causes delayed electrical conduction, precipitating re-entry phenomena adjacent to unaffected myocardium, finally fostering VT emanating from the RV. From a structural viewpoint, the replacement of healthy myocardium with fibro-fatty tissue compromises myocardial elasticity, contributing to both systolic and diastolic dysfunction. This alteration leads to a diminished cardiac ejection that cannot sustain myocardial demand, culminating in persistent ischemia and hypoxia.

In this case, the coronary angiography was initially performed to ascertain the etiology of the recurring VT episodes. The absence of significant coronary artery stenosis effectively excluded the possibility of coronary heart disease (CHD). The detection of epsilon waves on the admission ECG served as a critical marker for cardiomyopathy, particularly ARVC. Concurrently, echocardiography disclosed regional decreased wall motion in the RV and a pronounced decline in perfusion at the apical epicardium of the LV during MCE. This MCE detection may be rather unexpected for the reason that such pattern is in distinct from CHD, where perfusion decline commonly exhibits in the endocardial myocardium during MCE. The identification of diffuse stripy LGE in CMR explained the existence of myocardial fibrosis in the sub-epicardium of the left ventricular apex free wall. These findings, coupled with the clue of the RV enlargement in echocardiography and the hallmark epsilon wave on the ECG, substantiate the diagnosis of ARVC.

MCE is a non-invasive technique to assess myocardial microcirculation by means of ultrasound contrast agent (UCA) to display myocardial perfusion. The contrast echocardiography microbubbles are able to traverse the pulmonary circulation for their diminutive size smaller than capillaries, and then reach each segment of the myocardium. The echo intensity of UCA reflects the concentration of microbubbles within the myocardium, signifying the capillary blood volume when the UCA reaches the maximum saturation. Based on these attributes of UCA, enable a visual evaluation of myocardial microcirculation by examining the degree of microbubble replenishment, UCA clearance rate and the presence of perfusion defect. Microvascular dysfunction was determined when there was global or segmental lack of complete transmural or subendocardial microvascular refill within five cardiac cycles during vasodilator stress.

MCE is commonly used in clinical practice to detect myocardial ischemia and infarction in coronary artery disease, assess the establishment of collateral circulation, and evaluate myocardial viability. It is also used to observe intracardiac mass blood supply and perfusion, as well as to measure coronary flow reserve (stress echocardiography) to assess the effectiveness of interventional treatments. In this case, we observed the subepicardial ischemia in MCE, which suggests that it may serve as a valuable method for detecting myocardial abnormalities in ARVC patients. Moreover, it may provide as one of the differential diagnostic tools for differentiating between myocardial ischemia regarding CHD and myocardial intrinsic abnormalities. Indeed, confirming ARVC typically necessitates myocardial biopsy or CMR, which are either invasive or costly procedures. For patients with moderate suspicion of ARVC, direct progression to these diagnostic measures may not provide the most substantial diagnostic and therapeutic benefits, especially when access to such tools is limited in certain regions. The utilization of MCE is supposed to be considered as a helpful adjunctive tool for the diagnosis of ARVC in the mentioned circumstances.

## Conclusion

ARVC, as a rare primary cardiomyopathy, can be a diagnostic challenge leading to delayed treatment particularly for the young individuals. Therefore, it is imperative to conduct thorough examinations to ensure this uncommon condition is not overlooked, thereby averting delays in necessary interventions and management. In this instance, diminished subepicardial filling observed during MCE hints at its potential value in signaling myocardial anomalies associated with ARVC.

## Data Availability

All data and materials described in the manuscript are not publicly available due to the containing information that could compromise the privacy of patient. WL should be contacted if someone wants to request the data on reasonable request.

## Abbreviations

ARVC: Arrhythmogenic right ventricular cardiomyopathy
RV: Right ventricle
SCD: Sudden cardiac death
VT: Ventricular tachycardia
ECG: Electrocardiogram
HR: Heart rate
bpm: beats per minute
RFA: Radiofrequency ablation
TnT: Troponin T
PCT: Procalcitonin
LV: Left ventricle
LVO: Left ventricular opacification
MCE: Myocardial contrast echocardiography
CMR: Cardiovascular magnetic resonance
LGE: Late gadolinium enhancement
ICD: Intracardiac defibrillator
ITF: International Task Force
EMB: Endomyocardial biopsy
CHD: Coronary heart disease
UCA: Ultrasound contrast agent
VLMI: Very low mechanical index
